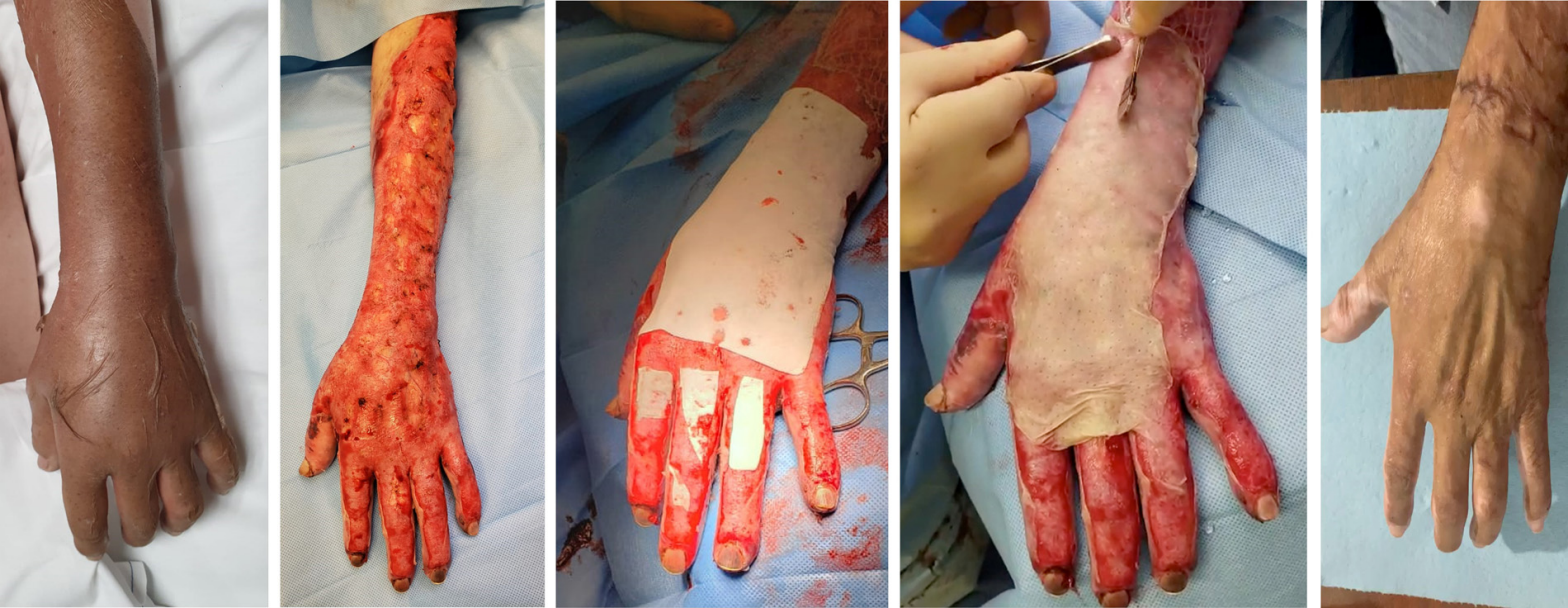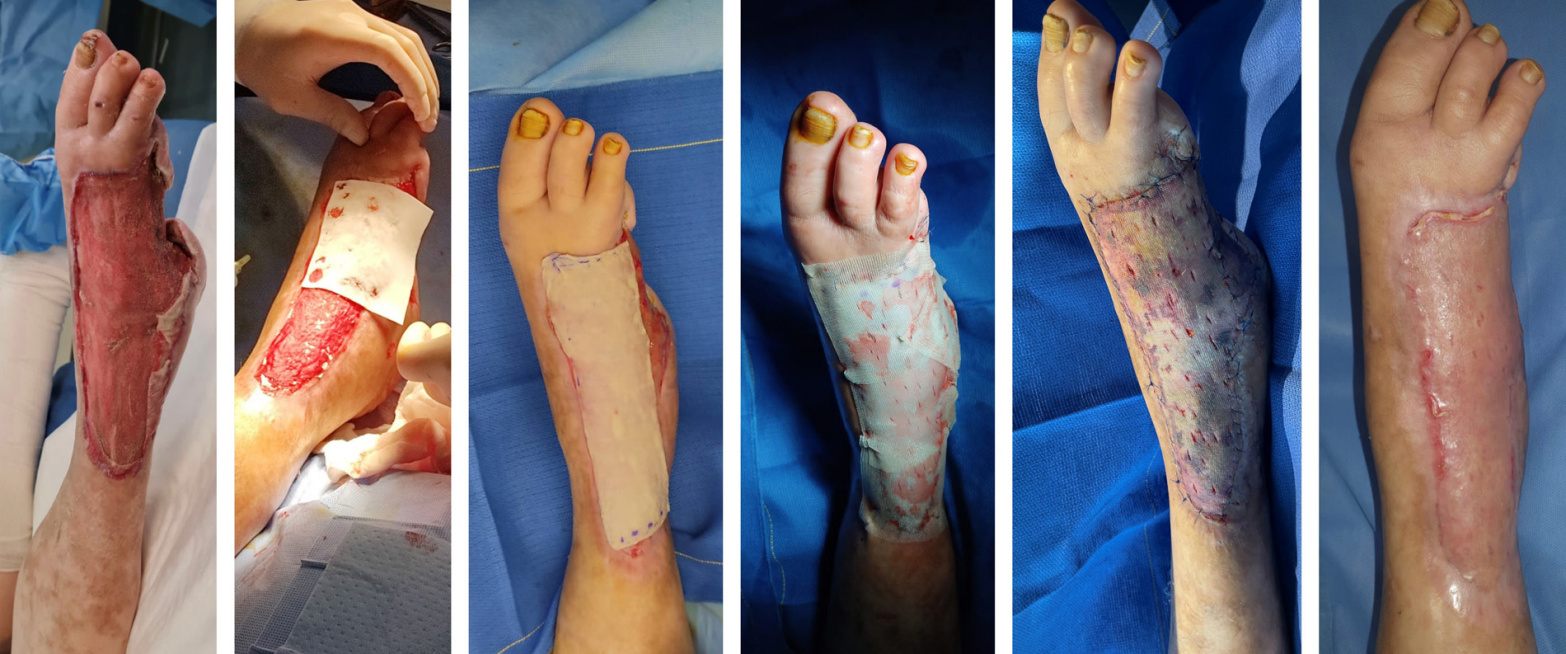# 558 Use of Dermal Matrix for Full Thickness Skin Defect Coverage

**DOI:** 10.1093/jbcr/iraf019.187

**Published:** 2025-04-01

**Authors:** Mauricio Manuel García-Pérez, Jorge Morales-Ortiz, Gabriel García-González, Mauricio Muñoz-Muñoz, Alejandro Quiroga-Garza

**Affiliations:** Hospital Universitario Dr. José Eleuterio González; Hospital Universitario Dr. José Eleuterio González; Hospital Universitario Dr. José Eleuterio González; Hospital General Dr. Norberto Treviño Zapata; Autonomous University of Nuevo León

## Abstract

**Introduction:**

Full-thickness skin defects caused by traumatic accidents, burns, tumor resections, and donor sites for free flaps should ideally be managed with full-thickness grafts. The ideal donor site is the groin area, but if the defect is of limited size, partial-thickness grafts may need to be used. The dermal matrix is made of bovine-derived type I, III, and V collagen and elastin, providing a structure that allows dermal cells to colonize it in an organized manner, thus fostering controlled dermal regeneration in combination with thin skin grafts.

**Methods:**

We present a series of cases using a dermal matrix of 1 mm combined with a thin partial-thickness skin graft (0.12 mm) in a single surgical procedure for the coverage of full-thickness defects.

**Results:**

CASE 1: A 28-year-old male with a history of direct flame burns on the left upper extremity (Fig. 5a) underwent debridement followed by the application of a dermal matrix and a partial-thickness skin graft.

CASE 2: A 52-year-old woman with type 2 diabetes presented with a history of an electrical burn injury that was managed with deep debridement with amputation and negative pressure therapy. Dermal matrixwas applied along with a partial-thickness skin graft.

**Conclusions:**

The use of dermal matrix in the treatment of full-thickness defects showed adequate dermal regeneration and coverage of defects in a single surgical procedure. Additionally, it restored elasticity in the new skin, reduced surgical risks, and shortened hospital stays compared to other more complex treatments.

**Applicability of Research to Practice:**

Dermal matrix improves the outcome of full-thickness skin grafts coverings on patients, resulting on a better skin quality in the long term, and could be taken into account intthe burn management algoeithms even before considering flaps.

**Funding for the Study:**

N/A